# Development of an innovative and sustainable one-step method for rapid plant DNA isolation for targeted PCR using magnetic ionic liquids

**DOI:** 10.1186/s13007-019-0408-x

**Published:** 2019-03-09

**Authors:** Arianna Marengo, Cecilia Cagliero, Barbara Sgorbini, Jared L. Anderson, Miranda N. Emaus, Carlo Bicchi, Cinzia M. Bertea, Patrizia Rubiolo

**Affiliations:** 1 Dipartimento di Scienza e Tecnologia del Farmaco, Università di Torino, Via P. Giuria 9, 10125 Turin, Italy; 2 Department of Chemistry, Iowa State University, Ames, IA 50011 USA; 3 Dipartimento di Scienze della Vita e Biologia dei Sistemi, Unità di Fisiologia Vegetale, Università di Torino, via Quarello 15/A, 10135 Turin, Italy

**Keywords:** DNA isolation, Magnetic ionic liquids, *Arabidopsis thaliana* (L.) Heynh., DNA barcoding

## Abstract

**Background:**

Nowadays, there is an increasing demand for fast and reliable plant biomolecular analyses. Conventional methods for the isolation of nucleic acids are time-consuming and require multiple and often non-automatable steps to remove cellular interferences, with consequence that sample preparation is the major bottleneck in the bioanalytical workflow. New opportunities have been created by the use of magnetic ionic liquids (MILs) thanks to their affinity for nucleic acids.

**Results:**

In the present study, a MIL-based magnet-assisted dispersive liquid–liquid microextraction (maDLLME) method was optimized for the extraction of genomic DNA from *Arabidopsis thaliana* (L.) Heynh leaves. MILs containing different metal centers were tested and the extraction method was optimized in terms of MIL volume and extraction time for purified DNA and crude lysates. The proposed approach yielded good extraction efficiency and is compatible with both quantitative analysis through fluorimetric-based detection and qualitative analysis as PCR amplification of multi and single locus genes. The protocol was successfully applied to a set of plant species and tissues.

**Conclusions:**

The developed MIL-based maDLLME approach exhibits good enrichment of nucleic acids for extraction of template suitable for targeted PCR; it is very fast, sustainable and potentially automatable thereby representing a powerful tool for screening plants rapidly using DNA-based methods.

**Electronic supplementary material:**

The online version of this article (10.1186/s13007-019-0408-x) contains supplementary material, which is available to authorized users.

## Background

Nowadays, there is an increasing demand for fast, sustainable, and reliable tools in plant biomolecular analyses. Plant DNA can be used for several fast screening applications including taxonomic studies [1], DNA barcoding for species discrimination and GMO and plant pathogen detection, for instance in food traceability or to verify the presence of contamination in food or herbal medicines [2–5].

High-quality DNA isolation is the first step in conducting plant molecular studies. However, with conventional methods, DNA extraction requires the use of toxic solvents and/or multiple and time-consuming steps to remove cellular interferences, such as polysaccharides, polyphenols, and other secondary metabolites, with the risk that DNA isolation can be the major bottleneck in the bioanalytical workflow. As a consequence, fast and cost-efficient DNA extraction protocols that yield high-quality DNA are highly desired in the study of species’ molecular genetics [6–8].

New perspectives have been realized by studying the interactions between DNA and ionic liquids (ILs) [9]. By definition, ILs are molten salts composed of organic or inorganic cations and anions that exhibit melting temperatures at or below 100 °C. Most ILs possess low or negligible vapor pressures under environmental conditions and are derived from bulky cations paired with weakly-coordinating anions. However, the choices of cation and anion structures are virtually limitless and result in the formation of ILs whose fundamental properties (including viscosity, conductivity, thermal stability, and hydrophobicity) can be customized for a given application [10]. A number of interactions between ILs and biological macromolecules have been shown with some studies even demonstrating that ILs are able to preserve the secondary or tertiary structure of biopolymers [11]. ILs were also successfully applied for the extraction of DNA from maize pellets; however, the protocol required the denaturation of the biopolymer for its recovery [12]. Magnetic ionic liquids (MILs) are an interesting subclass of ILs and possess all of the unique physico-chemical properties of ILs while also exhibiting strong susceptibility to external magnetic fields, due to the incorporation of a paramagnetic component in the cation and/or anion. A number of transition and rare earth metal centers have been previous explored including Fe(III), Mn(II), Gd(III), Ho(III), Dy(III), Co(II), and Ni(II) [13].

The use of hydrophobic MILs for the selective extraction and purification of nucleic acids was first demonstrated in 2015 [14]. Hydrophobic MILs can be used in magnet-assisted dispersive liquid–liquid microextraction (maDLLME) by dispersing the MILs into very small microdroplets within a water solution containing the nucleic acids followed by recovering the MILs with the application of a magnetic field. This approach results in reduced extraction times and increased extraction efficiency of the nucleic acids. The proposed extraction mechanism involves electrostatic interactions and ion exchange between the cation of the MIL with the negatively charged phosphate groups of nucleic acids. Furthermore, compared to other techniques exploiting magnetic fields such as magnetic beads or magnetic nanoparticles coated or functionalized with ILs, MILs can be easily prepared and are often optically transparent making them useful for spectroscopic applications. Until now, MILs have been employed for the extraction of plasmid DNA (pDNA), single-stranded DNA (ssDNA) and double-stranded (dsDNA) in either pure form, in complex solutions, or extracted from viable bacteria or plasma samples. However, to date no applications demonstrating the use of MILs as DNA extraction solvents in plants have been reported [15–18].

In this context, the aim of this study was to exploit MILs for the development of a reliable, fast, automatable and environmentally sensitive method for the isolation of DNA from plants that could be routinely applied for quantitative and qualitative biomolecular analyses, such as target end-point PCR and real-time PCR. Three low-viscosity hydrophobic MILs containing Co, Mn and Ni [19] metal centers were examined for the isolation of plant DNA. Preliminary tests were carried out on PCR products and purified genomic DNA both from *Arabidopsis thaliana* (L.) Heynh., whereas subsequent experiments were performed using plant material directly. Isolation of the DNA was carried out by using maDLLME after lysis of the plant cells followed by directly subjecting the DNA to qualitative and quantitative analyses. Finally, the protocol was successfully applied to a set of plant species and tissues.

## Results

### Evaluation of different extraction conditions for the isolation of the *A. thaliana ITS* PCR product

**Fig. 1:**
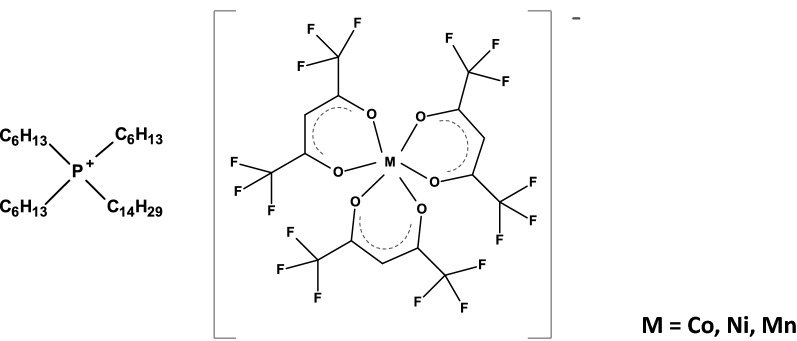
Chemical structures of the investigated MILs

The MIL-based approach using magnet-assisted dispersive liquid–liquid microextraction (maDLLME) of DNA was first tested on the PCR products of the internal-transcribed spacer (*ITS*) region of *A. thaliana.* The extraction performance was examined in terms of Enrichment Factor (ratio of the DNA concentration in the MIL and the DNA concentration in the initial solution) determined by fluorescence-based quantitation. MILs, each containing one of the three different metal centers investigated (i.e., Co, Ni and Mn) were assessed (Fig. 1) as was the influence of the different MIL volumes and extraction times.

**Fig. 2:**
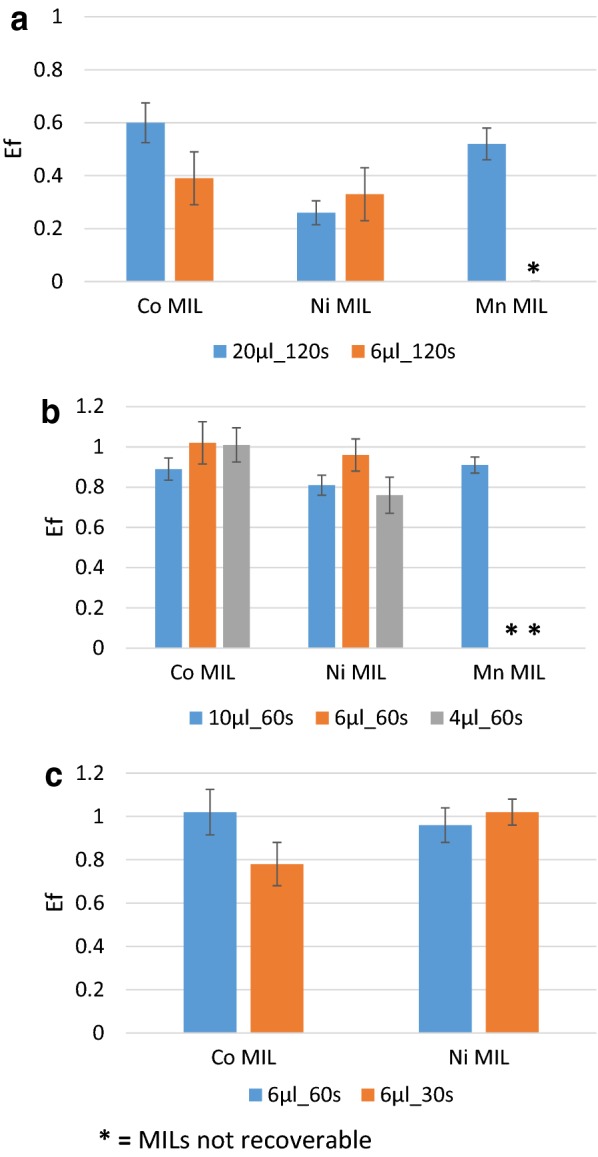
Enrichment Factors obtained after maDLLME under different conditions with the investigated MILs for the ITS PCR products of *A. thaliana*

Figure 2a shows the comparison between the Enrichment Factors for the three MILs obtained by dispersing 20 µl and 6 µl of the MIL in the buffer solution for 120 s. The results indicate that the Enrichment Factors were consistently below 1 for all of the investigated MILs and that the handheld magnet is not able to recover the Mn-containing MIL when a volume of 6 µl is used.

To decrease the solubility of the MILs in the buffer solution, the extraction time was subsequently reduced to 60 s. Figure 2b reports a comparison between the Enrichment Factors for the three MILs obtained by dispersing 10 µl, 6 µl and 4 µl of the MIL in the buffer solution for 60 s. The results show that a decrease in the extraction time resulted in a marked increase in the extraction efficiency with Enrichment Factors around 1 for all of the collected DNA-enriched MILs. Once again, the magnet was not able to recover the Mn-containing MIL when volumes below 10 µl are used; therefore, the Mn-containing MIL was excluded for the subsequent experiments. The performance of the Co and Ni-containing MILs were not significantly different (p > 0.05) with slightly better results obtained with the Co-containing MIL. A comparison of different MIL volumes also reveals very similar results with slightly higher Enrichment Factors obtained when using 6 µl of the MIL, particularly in the case of the Ni-containing MIL.

Finally, the possibility of further decreasing the extraction time was explored by testing the extraction efficiency at 30 s and comparing the results with those obtained at 60 s (Fig. 2c). The Enrichment Factor was not statistically different (p > 0.05) under the two conditions for both MILs demonstrating that the extraction time could be as fast as 30 s.

### Evaluation of different extraction conditions for the isolation of genomic DNA of *A. thaliana* from a water solution

**Fig. 3:**
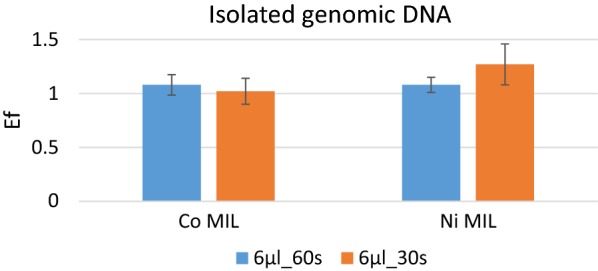
Enrichment Factors obtained after maDLLME under different conditions with the investigated MILs for genomic DNA from *A. thaliana* previously isolated with a commercial kit

The MIL-based extraction approach was then tested on the pure *A. thaliana* genomic DNA previously isolated with a commercial kit. The Co and Ni-containing MILs were tested; the MIL volume was fixed to 6 µl and 60 s and 30 s extraction times were applied. The results are reported in Fig. 3 and show good extraction efficiency (Enrichment Factors more than 1) for both MILs and extraction times.

### Evaluation of different conditions for the extraction of genomic DNA directly from *A. thaliana* plant material

**Fig. 4:**
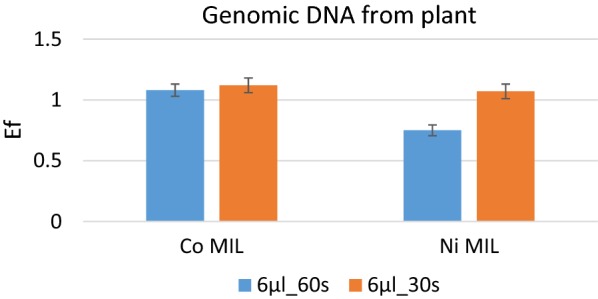
Enrichment Factors obtained after maDLLME under different conditions with the investigated MILs for genomic DNA from *A. thaliana* plant material after simple cell lysis

Finally, the proposed methodology was tested on *A. thaliana* genomic DNA directly from the plant cell lysate. Again, the Co and the Ni-containing MILs were tested and the MIL volume was fixed to 6 µl using extraction times of 60 s and 30 s. The Enrichment Factors determined by fluorimetric-based detection are reported in Fig. 4 and, again, the results show good extraction efficiency (Enrichment Factors more than 1) for both MILs. Slightly better results can be obtained with the Co-containing MIL, particularly when considering an extraction time of 60 s, the Enrichment Factor of the Ni-containing MIL under this condition was in fact statistically lower (p < 0.05).

### Compatibility of the extraction method with quantitative analysis

Quantitation of the isolated DNA was performed using a fluorescence-based detection approach directly on the DNA-enriched MILs using the Qubit Fluorometer (Life Technologies, Carlsbad, CA, USA). Compatibility of the fluorescence-based method with quantitative analysis was evaluated in two steps (see Table 1). Initially, the response of the pure MILs to the fluorometer was tested and no signal was detected for all MILs indicating that they didn’t interfere with the fluorimetric signal. Additionally, a solution containing the pure genomic DNA from *A. thaliana* was analysed as is and with the addition of 20 µl of each MIL in order to investigate any possible inhibition of the fluorimetric signal. As can be observed in Table 1, the concentration of DNA determined with the Co and Mn-containing MILs doesn’t differ from that determined in the pure DNA solution whereas a lower concentration was calculated if the Ni-containing MIL is added to the solution.

**Table 1:** Concentration of DNA in different solutions containing or not MILs determined by fluorimetric detection

	Blank solution (only buffer)	Test solution (solution with purified Genomic DNA from *A. thaliana*)
No MIL	< 0.2 ng/µl	0.491 ng/µl
Ni-containing MIL	< 0.2 ng/µl	0.249 ng/µl
Mn-containing MIL	< 0.2 ng/µl	0.531 ng/µl
Co-containing MIL	< 0.2 ng/µl	0.627 ng/µl

Instrumental limit of detection: 0.2 ng/µl

**Fig. 5:**
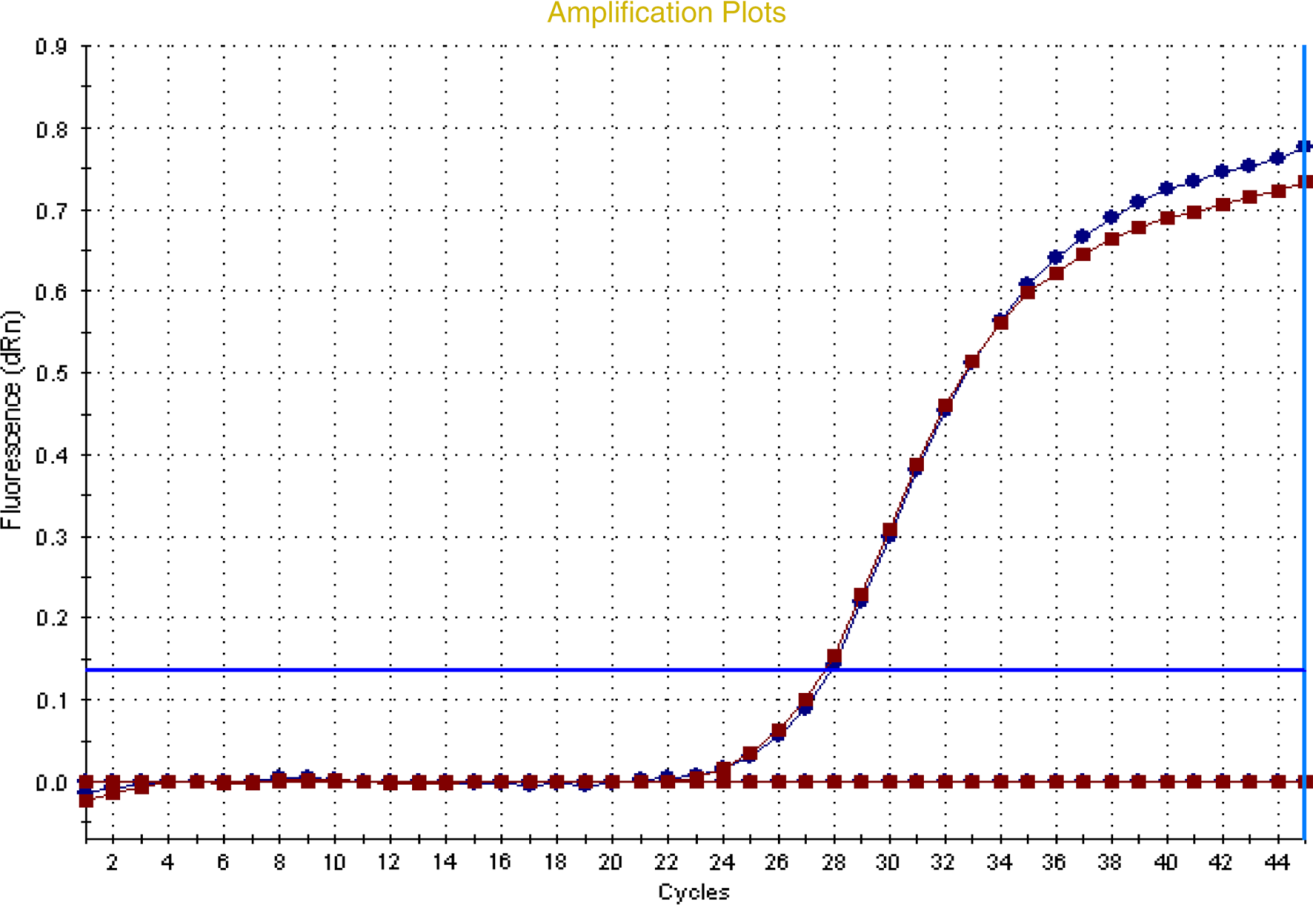
Amplification plots obtained by RT-PCR of the *AP2D* gene from *A. thaliana*. Blue (reference): DNA in the original sample (Ct: 24.23 min); red: DNA extracted with the maDLLLME approach and the Ni MIL (Ct: 24.11 min)

The extraction performance of the two investigated MILs was confirmed by real-time PCR. Figure 5 displays the curves for the Ni-containing MIL obtained by submitting the cell lysate and the DNA enriched MILs to real-time PCR. It is possible to notice that the curves are almost overlapped with very similar Ct values confirming that the Enrichment Factor is approximately one for both MILs under the optimized conditions.

The amount of DNA isolated with the Co-containing MIL from amplified short sequences, purified genomic DNA and genomic DNA from the plant cell lysate was also evaluated. The concentration of DNA was calculated directly in the DNA-enriched MILs with the fluorescence-based approach. The results are reported in Additional file 1: Table S1 and show that, considering the initial amount of DNA in each sample, the quantity of DNA extracted with the Co-containing MILs is 4.26, 0.302 and 2.92 ng/µl for the PCR products, the genomic DNA and the DNA extracted from *A. thaliana,* respectively. Similar results were obtained with the extraction carried out with the Ni-containing MILs: 4.1, 0.321 and 2.73 ng/µl for the PCR products, the genomic DNA and the DNA extracted from *A. thaliana*, respectively.

### Compatibility of the extraction method with qualitative analysis

The DNA-enriched MILs were also submitted to PCR amplification of specific sequences. Two different high copy noncoding intergenic spacers were selected in order to evaluate the possibility of applying the developed protocol, in particular, to barcoding investigations; single copy genes: *LFY* (encoding transcriptional regulator that promotes the transition to flowering), *FRI* (encoding for protein FRIGIDA), *BRC1* (encoding a TCP transcription factor), *MF* (encoding a Myb domain protein), *ASHH1* (encoding a SET domain-containing protein) were also selected [20].

**Fig. 6:**
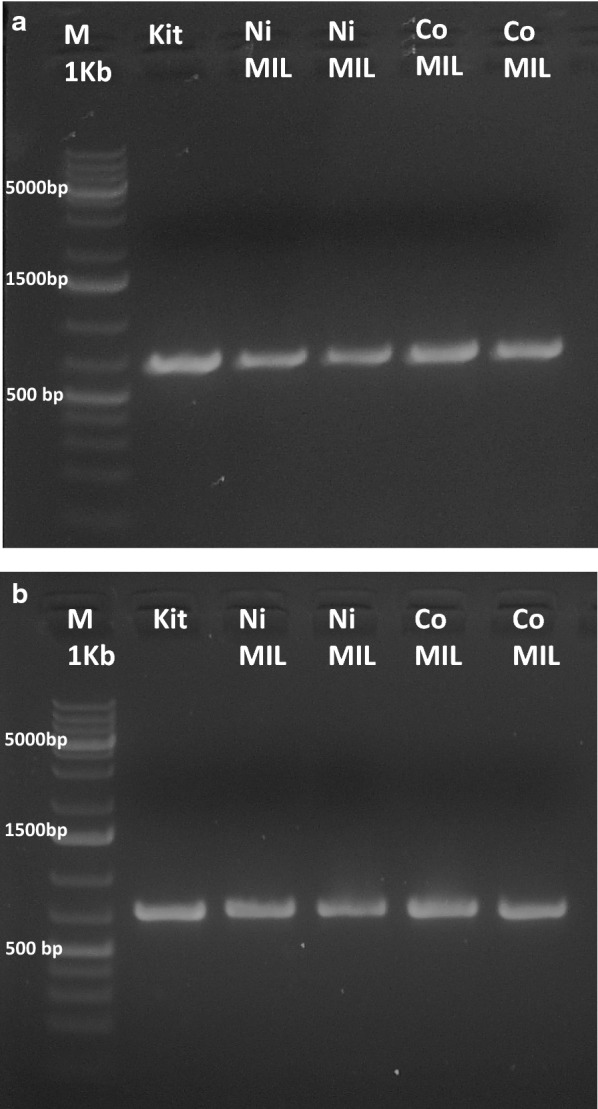
PCR amplification of the nuclear ribosomal DNA (nrDNA) internal transcribed spacer (*ITS*) sequence (**a**) and the intergenic spacers of the ribulose-1,5-bisphosphate carboxylase/oxygenase large subunit (*RbcL*) gene (**b**) from *A. thaliana*, after extraction with the commercial kit (lane 2), the Ni-containing MIL (lanes 3 and 4), and the Co-containing MIL (lane 5 and 6). The 1 Kb DNA ladder marker is shown in lane 1. Extraction conditions: 6 µl of MIL, extraction time: 30 s

The nuclear ribosomal DNA (nrDNA) internal transcribed spacer (*ITS*) sequence and the intergenic spacer of the ribulose-1,5-bisphosphate carboxylase/oxygenase large subunit (*RbcL*) were selected. The two regions were amplified by PCR using specific primers and the amplification products were submitted to agarose gel electrophoresis. The results are reported in Fig. 6a, b and show that with both the Co and Ni-containing MILs it is possible to successfully amplify the two investigated marker genes with short extraction times (30 s).

Good amplifications were also obtained on the five single copy genes: *LFY*, *FRI*, *BRC1*, *MF*, *ASHH1* [20, 21] that were amplified by PCR from the DNA isolated with the Co-containing MIL and then subjected to agarose gel electrophoresis (see Additional file 1: Figure S1).

Moreover, the longevity of the DNA was tested, the DNA from three samples of *A. thaliana* leaves was extracted with Co-containing MILs and stored for 20 days at room temperature. After this period, a back-extraction was performed from the collected MILs and the extracted DNA was subjected to PCR amplification, for both the multi locus *ITS* region and the single locus *FRI* region. A good amplification of both regions, using the stored MILs enriched DNA as template, was obtained (See Additional file 1: Figure S2).

### Application of the protocol to different plant species and tissues

The protocol was finally applied to the isolation of DNA from different plant tissues with high levels of impurities (polyphenols, polysaccharides, terpenoids, etc.). In particular, the genomic DNA from the leaves of *Solanum lycopersicum* L., *Perilla frutescens* (L.) Britton, *Stevia rebaudiana* (Bertoni) Bertoni and the roots of *Cucumis sativus* L. was extracted with Co-containing MILs. Since no results were obtained with 10 mg of starting plant material (data not shown), 5 mg of ground leaves or roots were used. Moreover, since 6 µl of Co-containing MILs often completely dissolved in the lysis solution (probably due to the presence of several interferences), 10 µl of Co-containing MILs were used for all the extractions, while the extraction time was maintained at 30 s. The obtained genomic DNA was subjected to PCR amplification, with primers specific for the *ITS* region. PCR products comparable to those of the commercial kit were obtained on the DNA isolated with the Co-containing MIL from the investigated plants, showing the potential application of the method to a wide number of species and to different plant tissues (see Additional file 1: Figure S3).

## Discussion

**Fig. 7:**
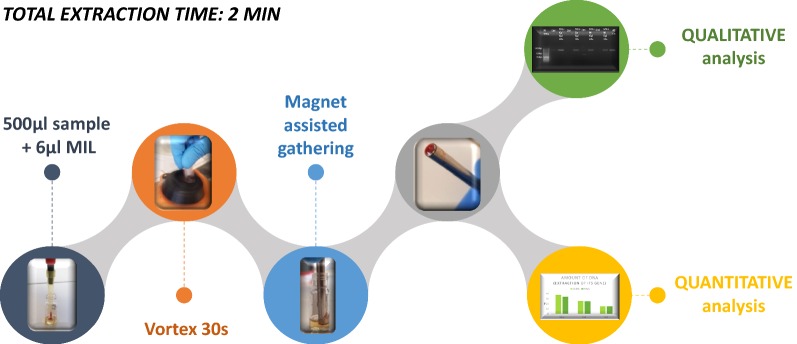
Flow-chart of the proposed extraction method

The present study proposes a new protocol for the extraction of genomic DNA from plants. A scheme of the optimized extraction protocol is reported in Fig. 7. The extraction step simply consists of the addition of the hydrophobic MILs into the aqueous sample solution. Due to the immiscibility of the MILs in the buffer solution, these compounds can be rapidly dispersed into small microdroplets that are able to bind the DNA [11]; afterwards, the DNA-enriched MILs can be collected by a handheld rod magnet. The collected MILs can be directly submitted to downstream analyses because of their compatibility with PCR [18] and with fluorescence-based quantitation methods (vide infra).

The first step of the present study was the optimization of the extraction conditions, considering *A. thaliana* (L.) Heynh as a model plant. The protocol was first set-up based on a short DNA sequence, in particular, we used the PCR products of the nuclear ribosomal DNA (nrDNA) internal transcribed spacer, *ITS* (~ 700 bp). MILs with different metal centers were employed using different extraction volumes and conducting extractions at different times. As shown in Fig. 2a, b, MILs with Co and Ni are suitable for the extraction of short sequences from plants, while the Mn-containing MIL did not give the same result in terms of recovery of the MIL microdroplets, especially when using low amounts of MIL. This poor result is probably related to its lower viscosity [19]. However, when considering the same extraction conditions, the performance of the Co and Ni-containing MIL are not statistically different (p > 0.005), with slightly higher extraction efficiency obtained for the Co-containing MIL. This result can be correlated to its more efficient recovery due to its higher magnetic susceptibility [19]. Considering the MIL volume, it is clear from Fig. 2a, b that higher extraction efficiencies can be obtained with lower amount of MILs; in particular, the optimal volume can be fixed at 6 µl, not only because of the higher value of Enrichment Factor, but also because of the higher absolute amount of DNA extracted. Concerning the extraction time, it can be observed from Fig. 2a, c that better results can be obtained with faster extraction times (30 or 60 s) since longer extraction times may increase the dissolution of MIL into the aqueous solution as it is evident by the very low extraction efficiency (Enrichment Factors far below 1) obtained by dispersing the MILs for 120 s. All of these results are in agreement with the results obtained by Emaus and co-workers who performed MIL-based extraction on the *KRAS* oncogene [18].

We then tested the extraction performance of the investigated MILs toward the genomic DNA of *A. thaliana* previously isolated with a commercial kit. The results were highly satisfactory with genomic DNA extraction efficiencies for the two MILs being comparable to those obtained with PCR products.

The final step of the optimization process was the evaluation of the extraction performance for the MILs in the presence of all interferences present in a plant cell lysate (primary and secondary metabolites). The extraction performance on the plant cell lysate was tested by both the determination of the Enrichment Factor by fluorimetric quantitation and through real-time PCR. As can be observed in Figs. 4 and 5, the MILs are able to extract DNA from a complex plant matrix with Enrichment Factors comparable to those obtained with pure DNA. Furthermore, the lysis buffer doesn’t interfere with the extraction efficiency, demonstrating that the proposed extraction protocol is applicable towards the isolation of nucleic acids directly from plant material after a very simple cell lysis reaction.

The amount of DNA isolated with the Co-containing MIL was also calculated and resulted in the order of some ng. This amount is not very high, but this result can be justified by the fact that the proposed protocol can be considered as a microextraction technique and a very low amount of chemicals (in our case magnetic ionic liquid) is required.

The low amount of DNA isolated with the proposed protocol makes very challenging a direct restriction digestion or an agarose gel detection; however, it was demonstrated that it is sufficient for all downstream analyses involving the nucleic acid amplification on both high copy numbers regions and single locus copy genes.

Moreover, the longevity of the DNA in the MILs and the selective extraction of DNA free of enzymes with nuclease activity, was successfully demonstrated with the PCR amplification of the DNA-enriched MILs stored for 20 days at room temperature, as it was previously shown by Clark et al. [14].

The extraction process is very fast and simple with an extremely reduced number of steps (Fig. 7). Furthermore, since the method does not require any centrifugation step and the extraction phase can be recovered with the application of a magnetic field, the entire process can be readily automated. Compared to conventional liquid–liquid extraction methods, the MIL-based approach doesn’t require the use of toxic solvents and is considerably faster. The method possesses several advantages compared to commercial plant DNA extraction kits including the fact that it is less expensive (approximately 0.0007 US$ per extraction with the MILs-based protocol compared to a price ranging from 0.9 to 2.4 US$ per extraction with the commercial standard kits) and more sustainable since it drastically reduces the amount of waste material and doesn’t require employing single-use plastic consumables.

The use of washable glass consumables makes this method more environmentally-friendly compared to conventional methods, but it can be disadvantageous because of the inclination of the MILs to interact with the glass vial instead of the magnet [18]. However, this problem can be easily overcome with the addition of a low amount of SDS surfactant used to promote cell wall lysis.

Some limitations in the use of MILs as extracting materials should also be considered. For example, the difficulty to evaluate of the DNA integrity (molecular weight/shearing) and quality (purity, suitability for further molecular biological procedures such as restriction enzyme digestion). Moreover, these compounds absorb in the UV range and quantitation of the extracted DNA cannot be performed through spectrophotometric techniques; therefore, fluorescence spectroscopy has to be used. Compatibility of the extraction method with fluorimetric quantitation was tested since it is has been reported in the literature that the paramagnetic ion of the MIL can quench fluorescence by nonradiative processes [22]. Our results confirmed that the Ni-containing MIL slightly inhibits the fluorescence signal while no inhibition was observed for the Mn and Co-containing MILs evaluated in this study. These data are in agreement with the previously reported study for only the Mn-containing MIL, while it was reported that the fluorescence intensity was quenched by the Co-containing MIL. However, this study employed a different fluorescence probe and only the Mn-containing MIL was tested in the presence of DNA into the tested solution. On the basis of our present results, we can therefore state that the Co-containing MIL is the optimal MIL not only in terms of good extraction performance but also in terms of its compatibility with downstream fluorimetric detection.

Finally, we have also demonstrated that the extraction method is applicable for DNA barcoding. DNA barcoding is a taxonomic method, supported by the Consortium for the Barcode of Life (CBOL), in which short DNA sequences selected for their inter-species variability and intra-species stability are used in order to identify an organism as belonging to a specific species. This is a useful method for species discrimination, for example, in food traceability or to verify the presence of contaminants in food or herbal medicines [2–5]. Within the barcoding genes normally used as bioidentification system for plants, *ITS* sequence and *RbcL* spacer were tested using *A. thaliana* genomic DNA as a template. Considering the fact that also the *RbcL* gene was successfully extracted and amplified, we have also demonstrated that the MIL-based extraction method is able to extract not only nuclear DNA but also plastidial DNA. Moreover, after the extraction of genomic DNA from the leaves of *S. lycopersicum* L., *P. frutescens* (L.) Britton, *S. rebaudiana* (Bertoni) Bertoni and the roots of *C. sativus* L., we successfully amplified the *ITS* barcoding sequence by PCR.

## Conclusions

In view of the continuous search for fast and cost-efficient DNA extraction protocols yielding high-quality DNA, this study explored the possibility of exploiting the unique properties of magnetic ionic liquids (MILs) in order to isolate DNA from plant matrices. The magnet-assisted dispersive liquid–liquid microextraction approach with three hydrophobic MILs was here tested for the first time on plants, in particular on short sequences, purified genomic DNA from *A. thaliana* (L.) Heynh and on direct-extraction of genomic DNA from the plant cell lysate of the leaves of *A. thaliana* (L.) Heynh, *S. lycopersicum* L., *P. frutescens* (L.) Britton and *S. rebaudiana* (Bertoni) Bertoni and the roots of *C. sativus* L.. Good results were obtained in terms of efficiency, particularly for the Co-containing MIL. The entire extraction method is simple, potentially automatable and fast since it takes only 2 min. Furthermore, it is compatible to both quantitative analysis (through fluorimetric-based detection) and qualitative analysis and requires the use of low amount of chemicals and of washable consumables making it highly sustainable.

MILs present new opportunities for the development of extraction approaches aimed at selectively binding specific DNA targets, in particular, barcoding genes. Ion-tagged oligonucleotides [23] and oligonucleotides possessing poly-cytosine tag [24] were in fact recently combined to a MIL support to successfully isolate specific sequences of DNA in the presence of interferences or from bacterial cell lysate. Based on the results obtained in the present study on the extraction of plant DNA with MILs, the applicability of the aforementioned selective extraction methods are under investigation by our group.

In plant molecular biology, the application of maDLLME with MILs represents a powerful tool for screening methods involving DNA, in particular for rapid extraction of template for targeted PCR. Considering the promising results obtained on the investigated plant matrices, in the future we will focus our attention on the adoption of the proposed protocol towards fast screening analyses (e.g. food barcoding, GMO and plant pathogen detection).

## Methods

### Chemicals

Manganese(II) chloride tetrahydrate was purchased from Alfa Aesar (Ward Hill, MA, USA). Nickel(II) chloride (98%), ammonium hydroxide (28–30% solution in water), and 1,1,1,5,5,5-hexafluoroacetylacetone (99%) were purchased from Acros Organics (Morris Plains, NJ, USA). Anhydrous diethyl ether (99.0%) was purchased from Avantor Performance Materials Inc. (Center Valley, PA, USA). Trihexyl(tetradecyl)phosphonium chloride (97.7%) was purchased from Strem Chemicals (Newburyport, MA, USA). Cobalt(II) chloride hexahydrate (98.0%) and sodium dodecyl sulfate (SDS) were purchased from Sigma-Aldrich (St. Louis, MO, USA). Neodymium rod and cylinder magnets (0.20 T, 0.66 T, and 0.9 T) were purchased from K&J Magnetics (Pipersville, PA, USA). The four MILs investigated in this study were synthesized and characterized using previously reported procedures [19]. The chemical structures of the four MILs are shown in Fig. 1. All MILs were purified using diethyl ether and water and subsequently dried in a vacuum oven overnight. When not in use, the MIL solvents were stored in a desiccator.

### Samples

The model plant used for the study was *A. thaliana* (L.) Heynh, Col 0. Seeds were washed with 1 mL of ethanol 75% for 2 min, then with 1 mL of sodium hypochlorite 5% for 5 min to sterilize their surface, and finally with sterile water for 3–4 times. Sterile agar plates (12  ×  12 cm), containing half-strength Murashige and Skoog (MS) medium, were prepared and used as support for the seed sown. After stratification for 48 h, plates were exposed horizontally under a white light source at 120 μmol m^−2^ s^−1^ and 21  °C (± 1.5), with a 16 h light/8 h dark photoperiod.

After approximately 2 weeks, plants were collected, dried at room temperature until constant weight and ground with a dry mortar and pestle to obtain a fine powder.

Three different *A. thaliana* samples were used to test the MIL DNA extraction ability:

- *ITS* PCR products (obtained as described in “PCR section”)
- isolated genomic DNA from *A. thaliana* (obtained as described in “DNA extraction with commercial kit” section)
- dried leaves from *A. thaliana* (obtained as previously described).

Further experiments on different species and plant tissues were performed. *Solanum lycopersicum* L., *P. frutescens* (L.) Britton and *S. rebaudiana* (Bertoni) Bertoni seeds were sown in plastic pots with sterilized potting soil and grown in a growth chamber at 23 °C and 60% humidity, photosynthetic photon flux rate (PPFR) of 120 μmol m^−2^ s^−1^ and a 16 h photoperiod [25]. The leaves were then collected, dried at room temperature until constant weight and ground with a dry mortar and pestle to obtain a fine powder. *Cucumis sativus* L. seeds were washed for 4 h with tap water, sown in quartz sand in plastic pots and grown in a growth chamber at 26/28 °C, 65% humidity, photosynthetic photon flux rate (PPFR) of 120 μmol m^−2^ s^−1^ and a 16 h photoperiod [26]. The roots were then collected, dried at room temperature until constant weight and ground with a dry mortar and pestle to obtain a fine powder.

### DNA extraction with MILs

For pure DNA samples (PCR products and pure genomic DNA), 400 μl of buffer (50 mM Tris–EDTA + 3 μM SDS) were added to 100 μl of sample in a 2 mL glass vial.

For the direct extraction from the leaves of *A. thaliana, S. lycopersicum*, *P. frutescens*, *S. rebaudiana* and the roots of *C. sativus* roots, a prior cell lysis reaction was performed. Ten mg of *A. thaliana* ground plant material and 5 mg of the other species plant tissues, together with approximately 5 mg of polyvinylpolypyrrolidone (PVPP, Sigma Aldrich, Bellefonte, USA), 15 μl of RNAse and 500 µl of a buffer solution containing 50 mM Tris–EDTA and 3 µM Sodium Dodecyl Sulfate (SDS) were mixed in a 1.5 mL microcentrifuge tube and incubated for 15 min at 100 °C. After 15 min of centrifugation at maximum speed the supernatant was collected and directly subjected to the extraction.

Four, 6, 10 or 20 μl of the Co, Ni, Mn MILs were added to approximately 500 µl of solution and dispersed for 30, 60 or 120 s in the aqueous phase using a vortex. The DNA-enriched MILs were then gathered in a single droplet with the aid of an external magnet, collected in a glass vial for analysis and directly submitted to quantification. To perform the PCR amplification a back-extraction was done: 30 µl of water were added to the DNA enriched MILs, the solution was heated at 90 °C for 10 min and an aliquot of 20 µl was then stored for downstream analyses [23].

To test the longevity of the DNA obtained with the MILs, three *A. thaliana* samples were extracted with Co-containing MILs and stored for 20 days at room temperature before back-extraction and PCR analysis.

All extractions were performed in triplicates.

### DNA extraction with commercial kit

Ten milligrams of ground leaves of *A. thaliana, S. lycopersicum*, *P. frutescens*, *S. rebaudiana* and roots of *C. sativus*, with the addition of approximately 5 mg of polyvinylpolypyrrolidone (PVPP, Sigma Aldrich, Bellefonte, USA) were used for the genomic DNA isolation using the NucleoSpin Plant II commercial kit (Macherey–Nagel, Düren, Germany) following the manufacturer’s instructions.

### PCR and gel electrophoresis

Genomic DNA extracted by the MILs was used as a template for PCR amplification, along with DNA isolated with the commercial kit. Additional file 1: Table S2 reports the forward and reverse primers specific for the amplification of *ITS*,* RbcL, LFY*, *FRI*, *BRC1*, *MF*, and *ASHH1* regions [27, 28]. The amplification was carried out in 25 μL reaction mixture containing: 2.5 μL of 10 × PCR buffer, 0.2 mM deoxynucleotide triphosphates (dNTPs) (Thermo-Scientific, Waltham, MA USA), 20 pmol of forward and reverse primers (Integrated DNA Technologies, BVBA, Leuven, Belgium), and 0.5 U of *Taq* DNA polymerase (Thermo- Scientific, Waltham, MA USA). PCR reactions were carried out in a T- Gradient Thermalcycler (Biometra GmbH, Gӧttingen, Germany). Cycling conditions consisted of an initial 4 min at 94 °C, followed by 30 s of denaturing at 94 °C, 45 s of annealing at 53 °C and 45 s of elongation at 72 °C, repeated for 35 cycles and with 10 min of final extension at 72 °C. PCR products were separated by 1.5% (w/v) agarose gel electrophoresis and visualized by ethidium bromide staining under UV.

### Quantitative real-time PCR

Quantitative real-time PCR (qPCR) was used to further verify the extraction efficiency of the investigated MILs. The forward primer AP2DF (5**′-**CTCAACTTCCCCTTTGTGGA-3′) and the reverse primer AP2DR (5′-CATATTGCAATCCCCTCCTC-3′) were used for the amplification of the AP2D (AP2 domain At4g34410) region (the primers were designed using Primer 3 software) [21, 29]. Analyses were performed in triplicate on DNA samples and control samples which included non-template controls to confirm the absence of contamination. All experiments were performed on a Stratagene Mx3000P™ Real-Time PCR System (Agilent Technologies, Santa Clara, CA USA) using SYBR Green I with ROX as an internal loading standard. The real-time PCR amplification was performed by using 0.6 μl of DNA with 0.3 μl of 10 μM primers, 5 μl of 2X Maxima™ SYBR green qPCR Master mix (Thermo Fisher, Waltham, MA USA) and sterile water up to 10 μl total volume. PCR conditions were as follow: 95 °C for 10 min, 95 °C for 20 s, 57 °C for 30 s, 72 °C for 35 s, 95 °C for 1 min, 55 °C for 30 s and 95 °C for 30 s. Fluorescence was read following each annealing and extension phase. All runs were followed by a melting curve analysis from 55 to 95 °C.

All amplification plots were analysed with MX3000P™ software to obtain Ct values.

### Quantitation by fluorescence-based detection and evaluation of the extraction performances

For each experiment, DNA quantification was performed through fluorimetric assay by using the Qubit 3.0 Fluorometer (Life Technologies, Carlsbad, CA, USA). Three μl of each sample was examined using the Qubit dsDNA HS Assay Kit 0.2–100 ng/μl (Life Technologies, Carlsbad, California, US) according to manufacturer’s instructions.

Considering the amount of DNA extracted by the MILs and the DNA present in the solution prior the MILs extraction, the Enrichment Factor (E_f_) was calculated for each MIL-based extraction, as reported in Eq. (1) (C_MIL_ = concentration of DNA extracted with the MILs; C_Std_ = concentration of the DNA prior to extraction) [18].

1 $${\text{E}}_{\text{f}} = {\text{ C}}_{\text{MIL}} /{\text{C}}_{\text{Std}}$$

## Additional file

**Additional file 1.** Supplementary data, i.e. PCR amplifications of single locus genes, stored DNA and different plant species and tissues, amounts of DNA after MILs extraction and list of primers used for PCR amplification.
