# Supplementary material for: Development of an innovative and sustainable one-step method for rapid plant DNA isolation for targeted PCR using magnetic ionic liquids
Source: Plant Methods. 2019 Mar 9;15:23. doi: 10.1186/s13007-019-0408-x (PMC6408755; doi:10.1186/s13007-019-0408-x)
Supplement: Supplementary file 1 — Additional file 1. Supplementary data, i.e. PCR amplifications of single locus genes, stored DNA and different plant species and tissues, amounts of DNA after MILs extraction and list of primers used for PCR amplification. [file 13007_2019_408_MOESM1_ESM.docx]

**SUPPLEMENTARY MATERIAL**

**DEVELOPMENT OF AN INNOVATIVE AND SUSTAINABLE ONE-STEP METHOD FOR RAPID PLANT DNA ISOLATION FOR TARGETED PCR USING MAGNETIC IONIC LIQUIDS**

Arianna Marengo, Cecilia Cagliero*, Barbara Sgorbini, Jared L. Anderson, Miranda N. Emaus, Carlo Bicchi, Cinzia M. Bertea, Patrizia Rubiolo

AUTHORS’ DETAILS

AM: Dipartimento di Scienza e Tecnologia del Farmaco, Università di Torino, Via P. Giuria 9, I-10125 Torino, Italy, email: [arianna.marengo@unito.it](mailto:arianna.marengo@unito.it)

CC: Dipartimento di Scienza e Tecnologia del Farmaco, Università di Torino, Via P. Giuria 9, I-10125 Torino, Italy, email: [cecilia.cagliero@unito.it](mailto:cecilia.cagliero@unito.it)

BS: Dipartimento di Scienza e Tecnologia del Farmaco, Università di Torino, Via P. Giuria 9, I-10125 Torino, Italy, email: [barbara.sgorbini@unito.it](mailto:barbara.sgorbini@unito.it)

JLA: Department of Chemistry, Iowa State University, Ames, Iowa 50011 (USA), email: [andersoj@iastate.edu](mailto:andersoj@iastate.edu)

MNE: Department of Chemistry, Iowa State University, Ames, Iowa 50011 (USA), email: [memaus@iastate.edu](mailto:memaus@iastate.edu)

CB: Dipartimento di Scienza e Tecnologia del Farmaco, Università di Torino, Via P. Giuria 9, I-10125 Torino, Italy, email: [carlo.bicchi@unito.it](mailto:carlo.bicchi@unito.it)

CMB: Dipartimento di Scienze della Vita e Biologia dei Sistemi, Unità di Fisiologia Vegetale, Università di Torino, via Quarello 15/A, 10135 Torino, Italy, email: [cinzia.bertea@unito.it](mailto:cinzia.bertea@unito.it)

PR: Dipartimento di Scienza e Tecnologia del Farmaco, Università di Torino, Via P. Giuria 9, I-10125 Torino, Italy, email: [patrizia.rubiolo@unito.it](mailto:patrizia.rubiolo@unito.it)

* Corresponding author: Cecilia Cagliero, Dipartimento di Scienza e Tecnologia del Farmaco, Università di Torino, Via P. Giuria 9, I-10125 Torino, Italy, email: [cecilia.cagliero@unito.it](mailto:cecilia.cagliero@unito.it)


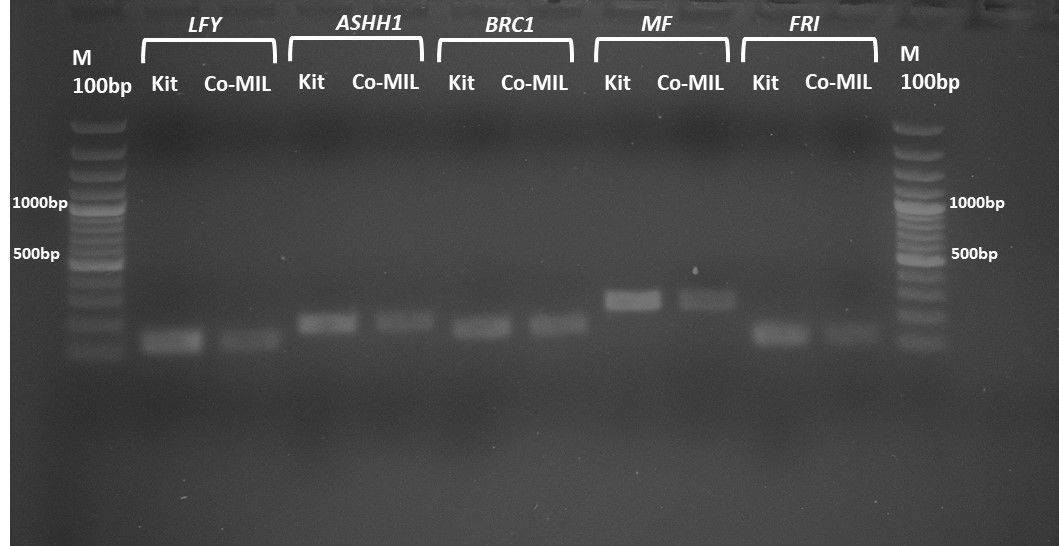


Fig.S1. PCR amplification of single locus genes from *A. thaliana* genomic DNA, after extraction with the commercial kit (lanes 2, 4, 6, 8, 10) and the Co-containing MILs (lanes 3, 5, 7, 9, 11). The 100bp ladder is shown in lanes 1 and 12. Co-containing MIL extraction conditions: 6 µl of MIL, extraction time: 30s.

*LFY* (encoding transcriptional regulator that promotes the transition to flowering)

*ASHH1* (encoding a SET domain-containing protein)

*BRC1* (encoding a TCP transcription factor)

*MF* (encoding a Myb domain protein)

*FRI* (encoding for protein FRIGIDA), BRC1 (encoding a TCP transcription factor)


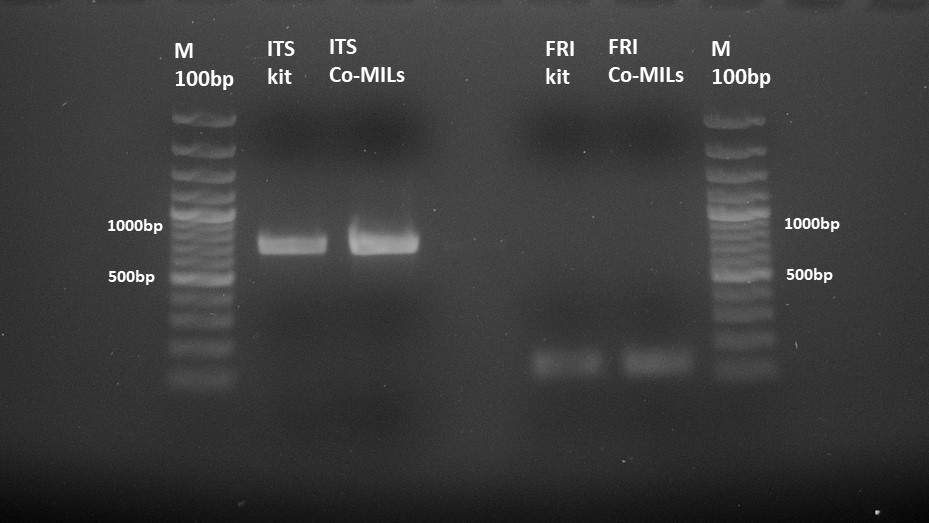


Fig.S2. PCR amplification of the nuclear ribosomal DNA (nrDNA) internal transcribed spacer (*ITS*) sequence (lanes 2 and 3) and the *FRI* single locus (lanes 4 and 5) from *A. thaliana* genomic DNA extracted with Co-containing MILs and stored for 20 days at room temperature and *A. thaliana* genomic DNA extracted with the commercial kit. The 100bp DNA ladder is shown in lanes 1 and 6. Co-containing MIL extraction conditions: 10 µl of MIL, extraction time: 30s.


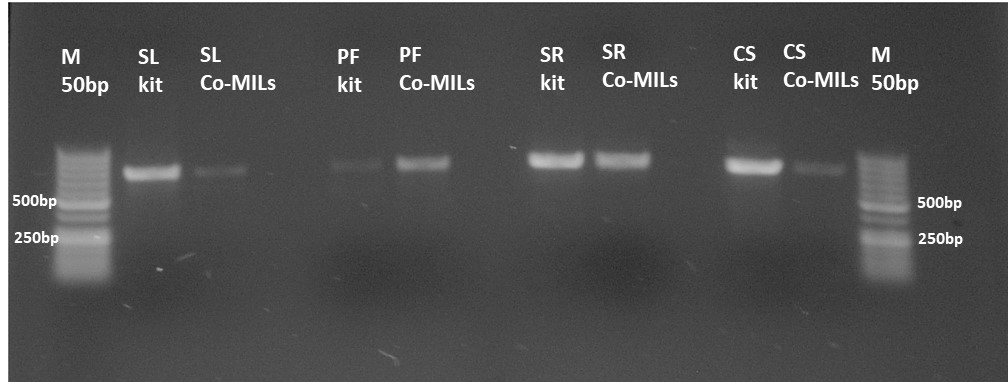


Fig.S3. PCR amplification of the nuclear ribosomal DNA (nrDNA) internal transcribed spacer (*ITS*) sequence from different plant species and tissues, after extraction with the commercial kit (lanes 2, 4, 6, 8), the Co-containing MILs (lanes 3, 5, 7, 9). The 50bp DNA ladder is shown in lanes 1 and 13. Co-containing MIL extraction conditions: 10 µl of MIL, extraction time: 30s.

SL: *Solanum lycopersicum*

PF: *Perilla frutescens*

SR: *Stevia rebaudiana*

CS: *Cucumis sativus*

**Table S1.** Amounts of DNA obtained after the cobalt and nichel MIL extraction. MIL extraction conditions: 6 µl of MIL, extraction time: 30s.

| **MILs** | **PCR products** | | **Genomic DNA** | | **Plant DNA** | |
| --- | --- | --- | --- | --- | --- | --- |
|  | Initial concentration (ng/µl) | DNA extracted (ng/µl) | Initial concentration (ng/µl) | DNA extracted (ng/µl) | Initial concentration (ng/µl) | DNA extracted (ng/µl) |
| **Co-containing MILs** | 4.17 | 4.26 | 0.344 | 0.302 | 2.75 | 2.92 |
| **Ni-containing MILs** | 4.33 | 4.1 | 0.293 | 0.321 | 3.58 | 2.73 |

**TableS2.** List of primers used for PCR amplification. BRC1, MF and ASHH1 primers, both forward and reverse, were designed using Primer 3 software [1, 2].

| **Primers** | **Primers sequence (5’-3’)** | **Tm (°C)** | **Gene Code** | **References** |
| --- | --- | --- | --- | --- |
| ITS1 | TCCGTAGGTGAACCTGCGG | 61 |  | [3] |
| ITS4 | TCCTCCGCTTATTGATATGC | 55.3 |  | [3] |
| RbcL1F | ATGTCACCACAAACAGAAAC | 53.2 |  | [4] |
| RbcL724R | TCGCATGTACCTGCAGTAGC | 59.4 |  | [4] |
| LFY_F | GCTCTCCACTGCCTAGACGA | 58.5 | At5g61850 | [5] |
| LFY_R | CATGACGACAAGCGATGTTC | 54.2 | At5g61850 | [5] |
| FRI_F | ATGCCTGATCGTGGTAAAGG | 54.8 | At4g00650 | [5] |
| FRI_R | CGCAGCTAATCCTCCTTCAG | 55.3 | At4g00650 | [5] |
| BRC1_F | CACTGAGCCCTCGGAAACTAT | 56.5 | At3g18550 |  |
| BRC1_R | TCATCCTACGATCTCGTGTCC | 55.7 | At3g18550 |  |
| MF_F | CTGGAAGGCGGTATGTAGGA | 56.1 | At3g27340 |  |
| MF_R | ATTGCTCCCAAGCTCGTAGA | 56.0 | At3g27340 |  |
| ASHH1_F | TCGCTCAGAAGCATGTTGAC | 55.4 | At1g76710 |  |
| ASSH1_R | TGCTGCTTCCTTCTTCACCT | 56.5 | At1g76710 |  |

References

1. Koressaar T, Remm M. Enhancements and modifications of primer design program Primer3. Bioinformatics. 2007; 23(10):1289-91
2. Untergasser A, Cutcutache I, Koressaar T, Ye J, Faircloth BC, Remm M and Rozen SG. Primer3--new capabilities and interfaces. 2012. Nucleic Acids Research. 40(15):e115.
3. Marengo A, Maxia A, Sanna C, Bertea CM, Bicchi C, Ballero M, et al. Characterization of four wild edible *Carduus* species from the Mediterranean region via phytochemical and biomolecular analyses. Food Res Int. 2017;100:822-31.
4. Fay MF, Swensen SM, Chase MW.Taxonomic Affinities of *Medusagyne oppositifolia* (Medusagynaceae). Kew Bulletin 1997; 52, 111–120.
5. Agliassa C, Narayana R, Bertea CM, Rodgers CT, Maffei ME. Reduction of the Geomagnetic Field Delays *Arabidopsis thaliana* Flowering Time Through Downregulation of Flowering-Related Genes. Bioelectromagnetics. 2018; 39(5):361-74.
